# Beyond SMARCB1 Loss: Recent Insights into the Pathobiology of Epithelioid Sarcoma

**DOI:** 10.3390/cells11172626

**Published:** 2022-08-24

**Authors:** Elisa Del Savio, Roberta Maestro

**Affiliations:** Unit of Oncogenetics and Functional Oncogenomics, Centro di Riferimento Oncologico di Aviano (CRO Aviano) IRCCS, National Cancer Institute, 33081 Aviano, PN, Italy

**Keywords:** epithelioid sarcoma, soft tissue sarcoma, SMARCB1, SWI/SNF, PRC2 (Polycomb Repressive Complex 2), tazemetostat

## Abstract

Epithelioid sarcoma (ES) is a very rare and aggressive mesenchymal tumor of unclear origin and uncertain lineage characterized by a prevalent epithelioid morphology. The only recurrent genetic alteration reported in ES as yet is the functional inactivation of SMARCB1 (SWI/SNF-related matrix-associated actin-dependent regulator of chromatin subfamily B member 1), a key component of the SWI/SNF (SWItch/Sucrose Non-Fermentable) chromatin remodeling complexes. How SMARCB1 deficiency dictates the clinicopathological characteristics of ES and what other molecular defects concur to its malignant progression is still poorly understood. This review summarizes the recent findings about ES pathobiology, including defects in chromatin remodeling and other signaling pathways and their role as therapeutic vulnerabilities.

## 1. Introduction

Epithelioid sarcoma (ES) is a very rare (incidence ~0.02–0.05/100,000) mesenchymal neoplasm of uncertain lineage characterized by a prevalent epithelioid morphology [1]. It was first described by Enzinger in 1970 as a “peculiar form of sarcoma that has been repeatedly confused with a chronic inflammatory process, a necrotizing granuloma and a squamous cell carcinoma” [2]. It primarily affects adolescents and young adults, accounting for less than 1% of all sarcomas in adults and 4–8% of pediatric non-rhabdomyosarcomatous sarcomas [3,4]. 

ES is an aggressive tumor. Although the disease is localized at presentation in about half of cases, ES shows a high rate of recurrences (from 15% to 60% in different series) and metastasizes at distal sites in 30–50% of the cases. Unlike other sarcomas, but similar to carcinomas, ES metastasizes to lymph nodes, further emphasizing its bivalent nature [5,6,7,8,9,10,11,12,13,14,15,16]. The five-year overall survival of patients with primary, localized disease is approximately 60% [1,17,18], whilst the median survival of metastatic cases is 12–18 months [4,18,19]. Pediatric ES appear to have a better prognosis than adult forms [3,20,21].

Histopathologically, ES is typically composed of nodules of cells with epithelioid morphology characterized by the co-expression of mesenchymal and epithelial markers. Specifically, ES cells are positive for vimentin, EMA (epithelial membrane antigen), and low-molecular-weight cytokeratins (CK8 and CK19), whereas they are usually negative for high-molecular-weight cytokeratins (CK5 and CK6) [22,23]. Over 50% of ES are positive for CD34, which is useful in the differential diagnosis with carcinomas [22,24,25]. ERG is also commonly expressed, which may lead to a misclassification with endothelial tumors [26,27,28]. As better described below, the loss of SMARCB1/INI1 protein expression is an essential diagnostic marker of ES [29]. 

According to the 2020 WHO classification of tumors of soft tissues and bone, ES is classified into two distinct subtypes: the classic or conventional type (C-ES, also called the “distal type”) and the proximal type (P-ES, also called the “large-cell type”), first described in 1997 [24,29]. Originally, the terms distal and proximal indicated the anatomical localization of the tumor. Nowadays, the classification in C-ES and P-ES has histological significance, irrespective of the site of the tumor, although C-ES mainly occurs in the distal upper extremities and P-ES primarily develops in the proximal axial regions of the body. A common feature of C-ES is the presence of cellular nodules of epithelioid and spindle cells surrounding a central area of degeneration and/or necrosis that resembles a granuloma [29]. Instead, P-ES are composed of large epithelioid, carcinoma-like cells often showing rhabdoid features and growing in a multi-nodular, sheet-like pattern [29]. The two ES variants differ also for demographic and clinical characteristics. C-ES is mainly observed in adolescents and young adults (20–40 years of age), with males being affected twice as often as females. C-ES presents as superficial, slowly growing and often ulcerated nodules that resemble a cutaneous squamous cell carcinoma [30], mostly affecting the distal upper extremities (hand, forearm and arm) [31]. P-ES is most frequently diagnosed in young to middle-aged adults (20–65 years of age), with a male-to-female ratio of 1.6:1. Deep soft tissues of proximal limbs, limb girdles, and midline of the trunk are the most common sites of P-ES development [24]. P-ES is considered more aggressive than C-ES, due to the higher rate of recurrences and earlier development of metastases [24,32]. 

## 2. SMARCB1 Loss, an ES Molecular Hallmark

A molecular hallmark of ES is the loss of SMARCB1/INI1 protein expression, although extremely rare cases compatible with ES diagnosis but retaining SMARCB1/INI1 immunostaining (SMARCB1-proficient ES) are described in the literature [29]. SMARCB1 (SWI/SNF-related matrix-associated actin-dependent regulator of chromatin subfamily B member 1), a.k.a. INI1 (Integrase Interactor 1), is a subunit of the mammalian SWI/SNF (SWItch/Sucrose Non-Fermentable) ATP-dependent chromatin remodeling complexes. SWI/SNF complexes, also known as BRG1/BRM-associated factor (BAF) complexes, are central regulators of nucleosome remodeling. By promoting sliding or ejection of nucleosomes, they facilitate the access of the transcriptional machinery to DNA [33,34,35]. 

**Figure 1:**
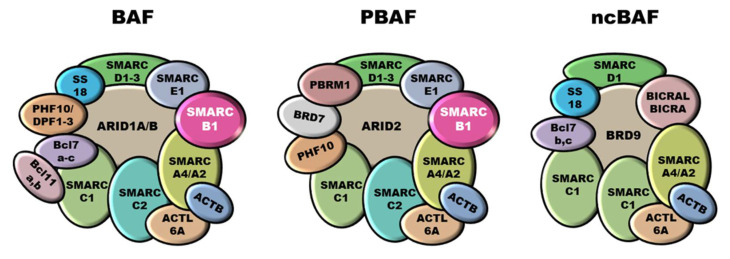
SWI/SNF chromatin remodeling complexes. SWI/SNF complexes are classified into three subgroups: canonical BAF (cBAF), polybromo-associated BAF (PBAF), and non-canonical BAF (ncBAF). SMARCB1 participates into cBAF and PBAF complexes whilst is not included into the ncBAF ones.

Mammalian SWI/SNF complexes are classified into three subgroups: canonical BAF (cBAF), polybromo-associated BAF (PBAF), and non-canonical BAF (ncBAF), also called GLTSCR1 or GLTSCR1L-containing and BRD9-containing (GBAF) complexes [36,37] (Figure 1). Some subunits and the ATPases are shared between all three subfamilies (e.g., SMARCC1, SMARCC2, SMARCD1, SMARCA4, SMARCA2), whereas other components are specific for each subgroup. SMARCB1 participates only into cBAF and PBAF complexes [38,39]. SWI/SNF complexes are involved in numerous biological processes, including cell cycle regulation and maintenance of genomic stability [40,41,42], and it has been estimated that alterations in SWI/SNF subunits involve over 20% of all cancers [42,43]. In particular, SMARCB1 is involved in ES, but also in malignant rhabdoid tumors (MRT), atypical teratoid/rhabdoid tumors (AT/RT) of the central nervous system, malignant peripheral nerve sheath tumors (MPNST), myoepithelial neoplasms and renal medullary carcinomas (RMC) [19,44,45,46]. 

SMARCB1 maps to chromosome 22q11. Although, in general, ES features a complex karyotype, with several numerical and structural alterations [47,48,49,50,51,52,53,54], chromosome defects involving 22q have been reported since the 1990s [48,51]. In 2005, Modena and coworkers, by combining spectral Karyotyping, FISH and CGH, identified SMARCB1 as the main target of these chromosome defects [55]. About 90% of ES harbor biallelic SMARCB1 deletions [56,57], which account for their negative SMARCB1/INI1 immunostaining. Rare nonsense frameshift and splice site mutations have been described as a source of SMARCB1/INI1 loss of expression [58]. Inactivating SMARCB1 deletions may be wide and involve neighboring genes such as BCR, which has been reported to be deleted in about 50% of ES [58], and EWSR1 (EWS RNA Binding Protein 1). The vicinity of SMARCB1 and EWSR1 may be source of misdiagnosis. In fact, a large deletion of SMARCB1 with involvement of EWSR1 may be misinterpreted as suggestive of EWSR1 rearrangement. In these cases, a detailed clinicopathologic correlation and in-depth genetic analyses are mandatory for correct diagnosis [59].

Very rare ES associated with hereditary, cancer predisposing SMARCB1 alterations have been reported. A case of homozyogously deleted ES occurring in the setting of SMARCB1 constitutional deletion was reported by Le Loarer and coworkers in a 25-year-old ES patient without prior familial or personal history of cancer [57]. More recently, an ES arisen in a patient affected by a rhabdoid tumor predisposition syndrome due to a constitutional intron/exon SMARCB1 variant (c.501-1G > A), likely responsible for aberrant mRNA splicing, has been documented [60]. 

In the fraction of ES that, although negative for SMARCB1/INI1 immunostaining, lack obvious SMARCB1 genetic defects (SMARCB1-intact ES), it has been suggested that SMARCB1 functional inactivation may be achieved through epigenetic mechanisms. Promoter silencing was ruled out as no promoter methylation was detected in SMARCB1-intact ES [61,62], and decitabine treatment failed to restore SMARCB1/INI1 expression [62]. Instead, several miRNAs have been proposed as SMARCB1 epigenetic inactivators. Based on the differential expression between SMARCB1-intact ES and SMARCB1-deleted MRT samples, miR-193a-5p, miR-206, miR-381, and miR-671-5p were suggested to be possibly implicated in the inactivation of SMARCB1 this subset of ES [63,64,65]. Unfortunately, the capacity of these miRNAs to actually inhibit SMARCB1 in the context of ES cells was not confirmed in other studies [62]. Therefore, the mechanism underlying the loss of SMARCB1/INI1 expression in SMARCB1-intact ES remains to be clarified. 

Similar to what reported for SMARCB1-negative MRT cells [66,67,68], restoration of SMARCB1 expression in SMARCB1-deleted ES cells (i.e., the VA-ES-BJ cell line) induces cell cycle arrest and impairs anchorage-independent growth and cell migration, substantiating its tumor suppressive role [69]. Moreover, the reintroduction of SMARCB1 in MRT cells correlated with re-activation of tissue-specific lineage-determining genes, indicating that failure of differentiation underpins tumor development in a context of SMARCB1 deficiency [70]. It is worth mentioning that the silencing of other components of the SWI/SNF complex in ES and MRT cells further affects proliferation, indicating that, despite the loss of SMARCB1/INI1, these tumors retain a residual SWI/SNF activity [62].

**Figure 2:**
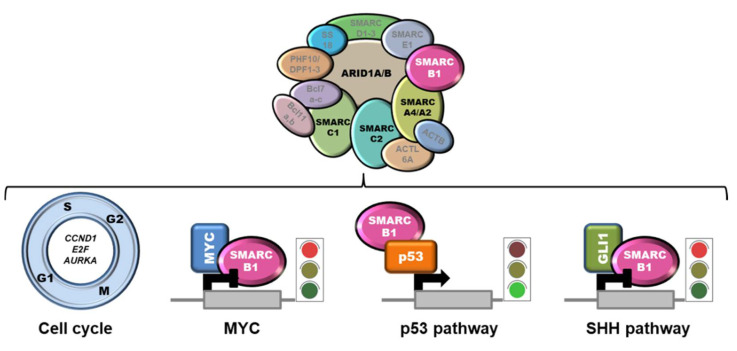
SMARCB1 intersection with relevant pathways. SMARCB1 negatively controls the expression of several cell cycle-related genes. Moreover, by interacting with MYC or GLI1, it hampers their transactivation activity. Conversely, the binding to p53 potentiates p53 tumor suppressive activity. The loss of the SWI/SNF subunits ARID1A, SMARCC1, SMARCC2, and SMARCA4 (in black) has been claimed to play a pathogenic role in the small fraction of SMARCB1-proficient ES.

The mechanism of action of SMARCB1 as a tumor suppressor relies on the intersection with several pathways, including cell proliferation and survival [71] (Figure 2). 

SMARCB1 negatively controls cyclin D1, E2F, and AURKA expression, and the loss of SMARCB1 in tumors was associated with an upregulation of these targets and cell cycle perturbation [68,72,73,74]. Moreover, SMARCB1 was demonstrated to directly bind MYC and to interfere with MYC-mediated transcriptional regulation [75,76]. Accordingly, SMARCB1 loss was correlated with enhanced MYC activity and increased DNA replication in diverse SMARCB1-negative cell lines, including MRT and ES cells [77]. Since BET bromodomain inhibitors have been shown to lead to repression of MYC-driven transcription [78], this class of compounds could represent a therapeutic avenue to be explored. SMARCB1 was reported to potentiate p53 transactivation activity by direct binding [79], and to favor nucleotide excision repair by interacting with several components of this machinery, including BRCA1, BARD1 and XPC [80,81]. This suggests that SMARCB1 deficiency may result in an alleviation of p53 tumor suppressive pathway and impaired control over genome stability. Functional interactions of SMARCB1 with Wnt/β-catenin and sonic hedgehog (SHH) signaling pathways have also been documented [82,83]. In particular, SMARCB1 has been shown to bind GLI1, a SHH effector, thereby inhibiting the SHH signaling. The loss of SMARCB1 results in GLI1 hyperactivation and increased tumorigenicity, a fact that has led to the hypothesis that GLI1 targeting may be therapeutic for SMARCB1-deficient tumors [82]. Finally, a role for SMARCB1 in regulating the expression of IL6 has been recently proposed [84].

**Figure 3:**
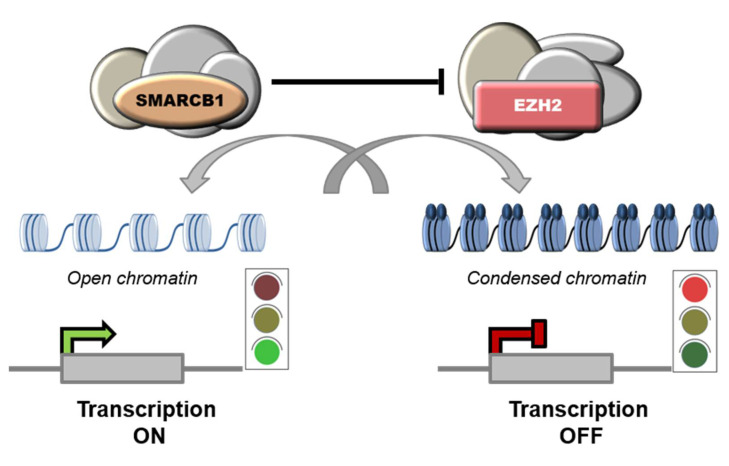
Functional antagonism between SWI/SNF and PRC2 complexes. SWI/SNF complexes regulate nucleosome remodeling by promoting sliding or ejection of nucleosomes, thus facilitating gene expression. Instead, PRC2 complexes induce chromatin compaction and transcriptional repression by catalyzing methylation of histone H3 on lysine 27 (H3K27).

Notably, SMARCB1 was shown to repress EZH2 and, accordingly, high levels of EZH2 have been reported in SMARCB1-deficient tumors, including ES [61], AT/RT [85], MRT [86] and chordomas [87]. EZH2 is the catalytic subunit of PRC2 complexes (Polycomb Repressive Complex 2) that mediate the transcriptional repression of target genes though methylation of histone H3 on lysine 27 (H3K27). [86]. PRC2 complexes are functional antagonists of the SWI/SNF complexes, and the balance between the two is key for cellular homeostasis [35,86,88,89] (Figure 3).

Concomitant inactivation of EZH2 and SMARCB1 is synthetic lethal: EZH2 silencing in SMARCB1-deficient MRT cells significantly impaired cell proliferation and triggered cell senescence in vitro and, in mouse models, it prevented the formation of tumors driven by SMARCB1 loss [86]. Similarly, EZH2 pharmacological inhibition induced strong anti-proliferative effects in SMARCB1-deleted cells and determined a complete regression of xenografts in mouse [90,91].

Based on these findings, EZH2-inhibitors have been considered as a potential therapeutic strategy in SMARCB1-deficient tumors. In 2013, an open-label, multicenter, phase I/II trial to evaluate the EZH2 inhibitor tazemetostat as a single agent in subjects with advanced solid tumors or with B-cell lymphomas was launched [92]. The study showed that tazemetostat had a safety profile and showed antitumor activity in a subset of patients that included ES. The study was followed by a phase II, multicenter study in adult subjects with SMARCB1/INI1-negative tumors or relapsed/refractory synovial sarcoma (NCT02601950) in 2015 [58]. Intriguingly, of the 62 ES patients enrolled in this trial, 15% (9/62) showed durable objective response after 32 weeks of treatment, and 21% (13/62) remained progression-free at 1 year [58]. These encouraging results led to the accelerated approval by the USA Food and Drug Administration (FDA) of tazemetostat for the treatment of adults and adolescents over 16 years of age with locally advanced or metastatic ES not eligible to complete surgical resection. In addition, based on preclinical evidence showing synergy between tazemetostat and doxorubicin [93], the therapeutic value of this combination is currently being evaluated as a frontline therapy in ES patients (NCT04204941). Other PRC2 components are also considered for therapeutic targeting. For instance, the clinical role of an inhibitor of EED (APG-5918/EEDi-5273), another core component of the PRC2 complexes, is going to be evaluated in different neoplasms, including ES (NCT05415098).

Intriguingly, a large fraction of SMARCB1/INI1-negative tumors show some degree of immune infiltration and PD-L1 expression [94,95], and EZH2 inhibition has been shown to have immunologic effects in both regulatory T cells and tumors [96]. These facts suggest that immune checkpoint inhibitors, as single agents or in combination with tazemetostat, may be promising therapeutic options. Accordingly, a number of clinical trials have been designed to address this hypothesis. For instance, a phase II clinical trial aimed to test the safety and effectiveness of the combination of nivolumab (anti-PD-1 antibody) and ipilimumab (anti-CTLA-4 antibody) in SMARCB1/INI1-negative tumors is ongoing (NCT04416568) and a phase I/II study to assess the value of combining these immune checkpoint inhibitors (nivolumab and ipilimumab) with tazemetostat has just been launched (NCT05407441) for the treatment of ES, MRT, AT/RT, chordoma and other SMARCB1/INI1- or SMARCA4-deficient tumors. An additional trial (NCT05286801) will determine the role of tiragolumab (anti-TIGIT antibody) and atezolizumab (anti-PD-L1 antibody) in relapsed or refractory SMARCB1- or SMARCA4-deficient tumors.

## 3. Other Players in ES Pathobiology

Beyond the loss of SMARCB1/INI1 expression, very little is known about the biology of ES. A Medline search was done using the following string “Epithelioid sarcoma AND (biology OR genetics)”. By 30 June 2022, 163 papers matched the search. The following section summarizes the main results of these works and the papers quoted therein.

Very few recurrent alterations have been detected as yet in ES, in addition to SMARCB1 inactivation. Functional inactivation of SMARCB1 seems to be paralleled by lack of expression of PBRM1/BAF180, another subunit of the SWI/SNF complexes. In fact, Li and coworkers reported that over 90% (17/18) of SMARCB1/INI1-negative ES fail to express also PBRM1/BAF180, suggesting a synergic role of these two proteins in ES pathogenesis [97]. Loss of the expression of other SWI/SNF subunits, namely SMARCA4/BRG1, SMARCC1/BAF155, SMARCC2/BAF170 [98], or ARID1A/BAF250A [99], has been claimed as causative for the extremely rare SMARCB1-proficient ES cases in which SMARCB1/INI1 protein expression is retained (Figure 2). Recently, amplification of BIRC3/YAP1 with trisomy of chromosome 2 was reported in an infantile SMARCB1-proficient ES [100], and a SS18-NEDD4 fusion was detected in a very aggressive cutaneous neoplasm with pathological characteristics resembling ES but retaining SMARCB1/INI1 expression [101]. It must be emphasized that it is still controversial whether SMARCB1-proficient ES actually exist or rather represent other, ES-like entities.

Besides impaired chromatin metabolism, ES features alterations in several signaling pathways, including EGFR, c-MET and AKT/mTOR. Cascio and colleagues reported strong and homogeneous membrane expression of EGFR in 73% (11/15) clinical ES samples analyzed by immunohistochemistry, although none of these cases showed evidence of EGFR amplification or activating mutations of the gene [102]. Marked expression of EGFR, associated with Tyr 1173 EGFR phosphorylation, was confirmed by Xie and colleagues. The authors also showed the involvement of the PI3K-AKT-mTOR signal transduction pathway in ES, with activation of mTOR (as inferred by the expression of the phosphorylated form of 4EBP1 and SRP) in all samples and loss of PTEN expression in 40% of the cases. In addition, they demonstrated that chemical inhibition of EGFR (erlotinib) impairs proliferation, migration, invasion and triggers apoptosis in ES cell models (Epi544 and VA-ES-BJ) [103,104]. Notably, the combination of EGFR blockade and mTOR inhibition (rapamycin) showed synergistic effects [103]. Synergistic effects were also observed for the combination of MET with EGFR [69] or mTOR inhibitors [69,104]. These effects were justified by the elevated levels of MET expression in both ES samples and cell lines [69,104]. 

ES also feature perturbation in the regulation of cell cycle. Jamshidi and colleagues demonstrated significantly reduced immunostaining for the CDK inhibitor p16 in about 1/3 of the samples analyzed [62]. The homozygous deletion of the CDKN2A locus, which encodes p16 and p14, was detected also in ES cell lines (VA-ES-BJ and HE-ES) [62,69]. Lualdi et al. reported copy number gain of the MYC gene in a large fraction of ES [54], but this result was not confirmed in other series [58,62]. 

Other mutations occasionally observed in ES include missense mutations in the putative tumor suppressor gene LRP1B [58], copy number gains of ABCA13, CAMK4 and KHDRBS2, heterozygous deletions of ERC1, NANOG, ING4, SSPN, TMTC1 and NF2 [62]. Overall, despite the apparent high mutation burden shown by ES [58,62] no specific mutation pattern beyond SMARCB1 loss has been identified so far, and even methylation profilings failed to identify recurrent patterns of biological significance in ES [58].

## 4. Conclusions

Despite numerous efforts to elucidate the genetics of ES, the only recurrent alteration detected to date in this very rare and aggressive sarcoma is the functional inactivation of SMARCB1. This suggests that ES is strongly driven by epigenetics. Characterizing the proteome of ES and better defining the molecular pathways specifically affected by the loss of SMARCB1 may help to define the molecular mechanisms of ES inception and progression and disclose novel therapeutic vulnerabilities.

## Data Availability

Not applicable.

## References

[1] Frezza A.M., Botta L., Pasquali S., Stacchiotti S., Gronchi A., Casali P.G., Trama A., Rarecarenet W.G. (2017). An Epidemiological Insight into Epithelioid Sarcoma (ES): The Open Issue of Distal-Type (DES) versus Proximal-Type (PES). Ann. Oncol..

[2] Enzinger F.M. (1970). Epitheloid Sarcoma. A Sarcoma Simulating a Granuloma or a Carcinoma. Cancer.

[3] Casanova M., Ferrari A., Collini P., Bisogno G., Alaggio R., Cecchetto G., Gronchi A., Meazza C., Garaventa A., Di Cataldo A. (2006). Epithelioid Sarcoma in Children and Adolescents: A Report from the Italian Soft Tissue Sarcoma Committee. Cancer.

[4] Jawad M.U., Extein J., Min E.S., Scully S.P. (2009). Prognostic Factors for Survival in Patients with Epithelioid Sarcoma: 441 Cases from the SEER Database. Clin. Orthop..

[5] Prat J., Woodruff J.M., Marcove R.C. (1978). Epithelioid Sarcoma: An Analysis of 22 Cases Indicating the Prognostic Significance of Vascular Invasion and Regional Lymph Node Metastasis. Cancer.

[6] Chase D.R., Enzinger F.M. (1985). Epithelioid Sarcoma. Diagnosis, Prognostic Indicators, and Treatment. Am. J. Surg. Pathol..

[7] Ross H.M., Lewis J.J., Woodruff J.M., Brennan M.F. (1997). Epithelioid Sarcoma: Clinical Behavior and Prognostic Factors of Survival. Ann. Surg. Oncol..

[8] Callister M.D., Ballo M.T., Pisters P.W., Patel S.R., Feig B.W., Pollock R.E., Benjamin R.S., Zagars G.K. (2001). Epithelioid Sarcoma: Results of Conservative Surgery and Radiotherapy. Int. J. Radiat. Oncol. Biol. Phys..

[9] Baratti D., Pennacchioli E., Casali P.G., Bertulli R., Lozza L., Olmi P., Collini P., Radaelli S., Fiore M., Gronchi A. (2007). Epithelioid Sarcoma: Prognostic Factors and Survival in a Series of Patients Treated at a Single Institution. Ann. Surg. Oncol..

[10] Wolf P.S., Flum D.R., Tanas M.R., Rubin B.P., Mann G.N. (2008). Epithelioid Sarcoma: The University of Washington Experience. Am. J. Surg..

[11] Guzzetta A.A., Montgomery E.A., Lyu H., Hooker C.M., Meyer C.F., Loeb D.M., Frassica D., Weber K.L., Ahuja N. (2012). Epithelioid Sarcoma: One Institution’s Experience with a Rare Sarcoma. J. Surg. Res..

[12] Levy A., Le Péchoux C., Terrier P., Bouaita R., Domont J., Mir O., Coppola S., Honoré C., Le Cesne A., Bonvalot S. (2014). Epithelioid Sarcoma: Need for a Multimodal Approach to Maximize the Chances of Curative Conservative Treatment. Ann. Surg. Oncol..

[13] Pradhan A., Grimer R.J., Abudu A., Tillman R.M., Carter S.R., Jeys L., Ferguson P.C., Griffin A.M., Wunder J.S. (2017). Epithelioid Sarcomas: How Important Is Loco-Regional Control?. Eur. J. Surg. Oncol..

[14] Outani H., Imura Y., Tanaka T., Takenaka S., Oshima K., Hamada K., Kakunaga S., Joyama S., Naka N., Kudawara I. (2018). Clinical Outcomes of Patients with Epithelioid Sarcomas: Impact and Management of Nodal Metastasis. Int. J. Clin. Oncol..

[15] Elsamna S.T., Amer K., Elkattawy O., Beebe K.S. (2020). Epithelioid Sarcoma: Half a Century Later. Acta Oncol..

[16] Frezza A.M., Sbaraglia M., Lo Vullo S., Baldi G.G., Simeone N., Frenos F., Campanacci D., Stacchiotti S., Pasquali S., Callegaro D. (2020). The Natural History of Epithelioid Sarcoma. A Retrospective Multicentre Case-Series within the Italian Sarcoma Group. Eur. J. Surg. Oncol..

[17] Jones R.L., Constantinidou A., Olmos D., Thway K., Fisher C., Al-Muderis O., Scurr M., Judson I.R. (2012). Role of Palliative Chemotherapy in Advanced Epithelioid Sarcoma. Am. J. Clin. Oncol..

[18] Frezza A.M., Jones R.L., Lo Vullo S., Asano N., Lucibello F., Ben-Ami E., Ratan R., Teterycz P., Boye K., Brahmi M. (2018). Anthracycline, Gemcitabine, and Pazopanib in Epithelioid Sarcoma: A Multi-Institutional Case Series. JAMA Oncol..

[19] Thway K., Jones R.L., Noujaim J., Fisher C. (2016). Epithelioid Sarcoma: Diagnostic Features and Genetics. Adv. Anat. Pathol..

[20] Sparber-Sauer M., Koscielniak E., Vokuhl C., Seitz G., Hallmen E., von Kalle T., Scheer M., Münter M., Bielack S.S., Ladenstein R. (2019). Epithelioid Sarcoma in Children, Adolescents, and Young Adults: Localized, Primary Metastatic and Relapsed Disease. Treatment Results of Five Cooperative Weichteilsarkom Studiengruppe (CWS) Trials and One Registry. Pediatr. Blood Cancer.

[21] Czarnecka A.M., Sobczuk P., Kostrzanowski M., Spalek M., Chojnacka M., Szumera-Cieckiewicz A., Rutkowski P. (2020). Epithelioid Sarcoma—From Genetics to Clinical Practice. Cancers.

[22] Miettinen M., Fanburg-Smith J.C., Virolainen M., Shmookler B.M., Fetsch J.F. (1999). Epithelioid Sarcoma: An Immunohistochemical Analysis of 112 Classical and Variant Cases and a Discussion of the Differential Diagnosis. Hum. Pathol..

[23] Laskin W.B., Miettinen M. (2003). Epithelioid Sarcoma: New Insights Based on an Extended Immunohistochemical Analysis. Arch. Pathol. Lab. Med..

[24] Guillou L., Wadden C., Coindre J.M., Krausz T., Fletcher C.D. (1997). “Proximal-Type” Epithelioid Sarcoma, a Distinctive Aggressive Neoplasm Showing Rhabdoid Features. Clinicopathologic, Immunohistochemical, and Ultrastructural Study of a Series. Am. J. Surg. Pathol..

[25] Fisher C. (2006). Epithelioid Sarcoma of Enzinger. Adv. Anat. Pathol..

[26] Miettinen M., Wang Z., Sarlomo-Rikala M., Abdullaev Z., Pack S.D., Fetsch J.F. (2013). ERG Expression in Epithelioid Sarcoma: A Diagnostic Pitfall. Am. J. Surg. Pathol..

[27] Stockman D.L., Hornick J.L., Deavers M.T., Lev D.C., Lazar A.J., Wang W.-L. (2014). ERG and FLI1 Protein Expression in Epithelioid Sarcoma. Mod. Pathol..

[28] Kohashi K., Yamada Y., Hotokebuchi Y., Yamamoto H., Taguchi T., Iwamoto Y., Oda Y. (2015). ERG and SALL4 Expressions in SMARCB1/INI1-Deficient Tumors: A Useful Tool for Distinguishing Epithelioid Sarcoma from Malignant Rhabdoid Tumor. Hum. Pathol..

[29] WHO Classification of Tumours Editorial Board (2020). Soft Tissue and Bone Tumours.

[30] Lin L., Skacel M., Sigel J.E., Bergfeld W.F., Montgomery E., Fisher C., Goldblum J.R. (2003). Epithelioid Sarcoma: An Immunohistochemical Analysis Evaluating the Utility of Cytokeratin 5/6 in Distinguishing Superficial Epithelioid Sarcoma from Spindled Squamous Cell Carcinoma. J. Cutan. Pathol..

[31] Spillane A.J., Thomas J.M., Fisher C. (2000). Epithelioid Sarcoma: The Clinicopathological Complexities of This Rare Soft Tissue Sarcoma. Ann. Surg. Oncol..

[32] Hasegawa T., Matsuno Y., Shimoda T., Umeda T., Yokoyama R., Hirohashi S. (2001). Proximal-Type Epithelioid Sarcoma: A Clinicopathologic Study of 20 Cases. Mod. Pathol..

[33] Kwon H., Imbalzano A.N., Khavari P.A., Kingston R.E., Green M.R. (1994). Nucleosome Disruption and Enhancement of Activator Binding by a Human SW1/SNF Complex. Nature.

[34] Reisman D., Glaros S., Thompson E.A. (2009). The SWI/SNF Complex and Cancer. Oncogene.

[35] Kadoch C., Copeland R.A., Keilhack H. (2016). PRC2 and SWI/SNF Chromatin Remodeling Complexes in Health and Disease. Biochemistry.

[36] Raab J.R., Resnick S., Magnuson T. (2015). Genome-Wide Transcriptional Regulation Mediated by Biochemically Distinct SWI/SNF Complexes. PLoS Genet..

[37] Alpsoy A., Dykhuizen E.C. (2018). Glioma Tumor Suppressor Candidate Region Gene 1 (GLTSCR1) and Its Paralog GLTSCR1-like Form SWI/SNF Chromatin Remodeling Subcomplexes. J. Biol. Chem..

[38] Michel B.C., D’Avino A.R., Cassel S.H., Mashtalir N., McKenzie Z.M., McBride M.J., Valencia A.M., Zhou Q., Bocker M., Soares L.M.M. (2018). A Non-Canonical SWI/SNF Complex Is a Synthetic Lethal Target in Cancers Driven by BAF Complex Perturbation. Nat. Cell Biol..

[39] Centore R.C., Sandoval G.J., Soares L.M.M., Kadoch C., Chan H.M. (2020). Mammalian SWI/SNF Chromatin Remodeling Complexes: Emerging Mechanisms and Therapeutic Strategies. Trends Genet. TIG.

[40] Medjkane S., Novikov E., Versteege I., Delattre O. (2004). The Tumor Suppressor HSNF5/INI1 Modulates Cell Growth and Actin Cytoskeleton Organization. Cancer Res..

[41] Vries R.G.J. (2005). Cancer-Associated Mutations in Chromatin Remodeler HSNF5 Promote Chromosomal Instability by Compromising the Mitotic Checkpoint. Genes Dev..

[42] Wang X., Haswell J.R., Roberts C.W.M. (2014). Molecular Pathways: SWI/SNF (BAF) Complexes Are Frequently Mutated in Cancer-Mechanisms and Potential Therapeutic Insights. Clin. Cancer Res..

[43] Kadoch C., Hargreaves D.C., Hodges C., Elias L., Ho L., Ranish J., Crabtree G.R. (2013). Proteomic and Bioinformatic Analysis of Mammalian SWI/SNF Complexes Identifies Extensive Roles in Human Malignancy. Nat. Genet..

[44] Hollmann T.J., Hornick J.L. (2011). INI1-Deficient Tumors: Diagnostic Features and Molecular Genetics. Am. J. Surg. Pathol..

[45] Agaimy A. (2014). The Expanding Family of SMARCB1(INI1)-Deficient Neoplasia: Implications of Phenotypic, Biological, and Molecular Heterogeneity. Adv. Anat. Pathol..

[46] Margol A.S., Judkins A.R. (2014). Pathology and Diagnosis of SMARCB1-Deficient Tumors. Cancer Genet..

[47] Molenaar W.M., DeJong B., Dam-Meiring A., Postma A., DeVries J., Hoekstra H.J. (1989). Epithelioid Sarcoma or Malignant Rhabdoid Tumor of Soft Tissue? Epithelioid Immunophenotype and Rhabdoid Karyotype. Hum. Pathol..

[48] Cordoba J.C., Parham D.M., Meyer W.H., Douglass E.C. (1994). A New Cytogenetic Finding in an Epithelioid Sarcoma, t(8;22)(Q22;Q11). Cancer Genet. Cytogenet..

[49] Iwasaki H., Ohjimi Y., Ishiguro M., Isayama T., Kaneko Y., Yoh S., Emoto G., Kikuchi M. (1996). Epithelioid Sarcoma with an 18q Aberration. Cancer Genet. Cytogenet..

[50] Sonobe H., Ohtsuki Y., Sugimoto T., Shimizu K. (1997). Involvement of 8q, 22q, and Monosomy 21 in an Epithelioid Sarcoma. Cancer Genet. Cytogenet..

[51] Quezado M.M., Middleton L.P., Bryant B., Lane K., Weiss S.W., Merino M.J. (1998). Allelic Loss on Chromosome 22q in Epithelioid Sarcomas. Hum. Pathol..

[52] Dal Cin P., Van den Berghe H., Pauwels P. (1999). Epithelioid Sarcoma of the Proximal Type with Complex Karyotype Including i(8q). Cancer Genet. Cytogenet..

[53] Debiec-Rychter M., Sciot R., Hagemeijer A. (2000). Common Chromosome Aberrations in the Proximal Type of Epithelioid Sarcoma. Cancer Genet. Cytogenet..

[54] Lualdi E., Modena P., Debiec-Rychter M., Pedeutour F., Teixeira M.R., Facchinetti F., Dagrada G.P., Pilotti S., Sozzi G. (2004). Molecular Cytogenetic Characterization of Proximal-Type Epithelioid Sarcoma. Genes Chromosomes Cancer.

[55] Modena P., Lualdi E., Facchinetti F., Galli L., Teixeira M.R., Pilotti S., Sozzi G. (2005). SMARCB1/INI1 Tumor Suppressor Gene Is Frequently Inactivated in Epithelioid Sarcomas. Cancer Res..

[56] Sullivan L.M., Folpe A.L., Pawel B.R., Judkins A.R., Biegel J.A. (2013). Epithelioid Sarcoma Is Associated with a High Percentage of SMARCB1 Deletions. Mod. Pathol..

[57] Le Loarer F., Zhang L., Fletcher C.D., Ribeiro A., Singer S., Italiano A., Neuville A., Houlier A., Chibon F., Coindre J.-M. (2014). Consistent SMARCB1 Homozygous Deletions in Epithelioid Sarcoma and in a Subset of Myoepithelial Carcinomas Can Be Reliably Detected by FISH in Archival Material. Genes Chromosomes Cancer.

[58] Gounder M., Schöffski P., Jones R.L., Agulnik M., Cote G.M., Villalobos V.M., Attia S., Chugh R., Chen T.W.-W., Jahan T. (2020). Tazemetostat in Advanced Epithelioid Sarcoma with Loss of INI1/SMARCB1: An International, Open-Label, Phase 2 Basket Study. Lancet Oncol..

[59] Huang S.-C., Zhang L., Sung Y.-S., Chen C.-L., Kao Y.-C., Agaram N.P., Antonescu C.R. (2016). Secondary EWSR1 Gene Abnormalities in SMARCB1-Deficient Tumors with 22q11-12 Regional Deletions: Potential Pitfalls in Interpreting EWSR1 FISH Results. Genes Chromosomes Cancer.

[60] Baker T.G., Lyons M.J., Leddy L., Parham D.M., Welsh C.T. (2021). Epithelioid Sarcoma Arising in a Long-Term Survivor of an Atypical Teratoid/Rhabdoid Tumor in a Patient with Rhabdoid Tumor Predisposition Syndrome. Pediatr. Dev. Pathol..

[61] Papp G., Changchien Y.-C., Péterfia B., Pecsenka L., Krausz T., Stricker T.P., Khoor A., Donner L., Sápi Z. (2013). SMARCB1 Protein and MRNA Loss Is Not Caused by Promoter and Histone Hypermethylation in Epithelioid Sarcoma. Mod. Pathol..

[62] Jamshidi F., Bashashati A., Shumansky K., Dickson B., Gokgoz N., Wunder J.S., Andrulis I.L., Lazar A.J., Shah S.P., Huntsman D.G. (2016). The Genomic Landscape of Epithelioid Sarcoma Cell Lines and Tumours. J. Pathol..

[63] Kohashi K., Yamamoto H., Kumagai R., Yamada Y., Hotokebuchi Y., Taguchi T., Iwamoto Y., Oda Y. (2014). Differential MicroRNA Expression Profiles between Malignant Rhabdoid Tumor and Epithelioid Sarcoma: MiR193a-5p Is Suggested to Downregulate SMARCB1 MRNA Expression. Mod. Pathol..

[64] Papp G., Krausz T., Stricker T.P., Szendrői M., Sápi Z. (2014). *SMARCB1* Expression in Epithelioid Sarcoma Is Regulated by MiR-206, MiR-381, and MiR-671-5p on Both MRNA and Protein Levels: *Smarcb1* Regulation By Mirnas In Epithelioid Sarcoma. Genes Chromosomes Cancer.

[65] Sápi Z., Papp G., Szendrői M., Pápai Z., Plótár V., Krausz T., Fletcher C.D.M. (2016). Epigenetic Regulation of *SMARCB1* By MiR-206, -381 and -671-5p Is Evident in a Variety of SMARCB1 Immunonegative Soft Tissue Sarcomas, While MiR-765 Appears Specific for Epithelioid Sarcoma. A MiRNA Study of 223 Soft Tissue Sarcomas. Genes Chromosomes Cancer.

[66] Ae K., Kobayashi N., Sakuma R., Ogata T., Kuroda H., Kawaguchi N., Shinomiya K., Kitamura Y. (2002). Chromatin Remodeling Factor Encoded by Ini1 Induces G1 Arrest and Apoptosis in Ini1-Deficient Cells. Oncogene.

[67] Betz B.L., Strobeck M.W., Reisman D.N., Knudsen E.S., Weissman B.E. (2002). Re-Expression of HSNF5/INI1/BAF47 in Pediatric Tumor Cells Leads to G_1_ Arrest Associated with Induction of P16ink4a and Activation of RB. Oncogene.

[68] Versteege I., Medjkane S., Rouillard D., Delattre O. (2002). A Key Role of the HSNF5/INI1 Tumour Suppressor in the Control of the G1-S Transition of the Cell Cycle. Oncogene.

[69] Brenca M., Rossi S., Lorenzetto E., Piccinin E., Piccinin S., Rossi F.M., Giuliano A., Dei Tos A.P., Maestro R., Modena P. (2013). *SMARCB1/INI1* Genetic Inactivation Is Responsible for Tumorigenic Properties of Epithelioid Sarcoma Cell Line VAESBJ. Mol. Cancer Ther..

[70] Kenny C., O’Meara E., Ulaş M., Hokamp K., O’Sullivan M.J. (2021). Global Chromatin Changes Resulting from Single-Gene Inactivation—The Role of SMARCB1 in Malignant Rhabdoid Tumor. Cancers.

[71] Cooper G.W., Hong A.L. (2022). SMARCB1-Deficient Cancers: Novel Molecular Insights and Therapeutic Vulnerabilities. Cancers.

[72] Lee S., Cimica V., Ramachandra N., Zagzag D., Kalpana G.V. (2011). *Aurora A* Is a Repressed Effector Target of the Chromatin Remodeling Protein INI1/HSNF5 Required for Rhabdoid Tumor Cell Survival. Cancer Res..

[73] Lin L., Hicks D., Xu B., Sigel J.E., Bergfeld W.F., Montgomery E., Fisher C., Hartke M., Tubbs R., Goldblum J.R. (2005). Expression Profile and Molecular Genetic Regulation of Cyclin D1 Expression in Epithelioid Sarcoma. Mod. Pathol..

[74] Isakoff M.S., Sansam C.G., Tamayo P., Subramanian A., Evans J.A., Fillmore C.M., Wang X., Biegel J.A., Pomeroy S.L., Mesirov J.P. (2005). Inactivation of the Snf5 Tumor Suppressor Stimulates Cell Cycle Progression and Cooperates with P53 Loss in Oncogenic Transformation. Proc. Natl. Acad. Sci. USA.

[75] Stojanova A., Tu W.B., Ponzielli R., Kotlyar M., Chan P.-K., Boutros P.C., Khosravi F., Jurisica I., Raught B., Penn L.Z. (2016). MYC Interaction with the Tumor Suppressive SWI/SNF Complex Member INI1 Regulates Transcription and Cellular Transformation. Cell Cycle.

[76] Weissmiller A.M., Wang J., Lorey S.L., Howard G.C., Martinez E., Liu Q., Tansey W.P. (2019). Inhibition of MYC by the SMARCB1 Tumor Suppressor. Nat. Commun..

[77] Msaouel P., Malouf G.G., Su X., Yao H., Tripathi D.N., Soeung M., Gao J., Rao P., Coarfa C., Creighton C.J. (2020). Comprehensive Molecular Characterization Identifies Distinct Genomic and Immune Hallmarks of Renal Medullary Carcinoma. Cancer Cell.

[78] Delmore J.E., Issa G.C., Lemieux M.E., Rahl P.B., Shi J., Jacobs H.M., Kastritis E., Gilpatrick T., Paranal R.M., Qi J. (2011). BET Bromodomain Inhibition as a Therapeutic Strategy to Target C-Myc. Cell.

[79] Lee D., Kim J.W., Seo T., Hwang S.G., Choi E.-J., Choe J. (2002). SWI/SNF Complex Interacts with Tumor Suppressor P53 and Is Necessary for the Activation of P53-Mediated Transcription. J. Biol. Chem..

[80] Ray A., Mir S.N., Wani G., Zhao Q., Battu A., Zhu Q., Wang Q.-E., Wani A.A. (2009). Human SNF5/INI1, a Component of the Human SWI/SNF Chromatin Remodeling Complex, Promotes Nucleotide Excision Repair by Influencing ATM Recruitment and Downstream H2AX Phosphorylation. Mol. Cell. Biol..

[81] Fontana G.A., Rigamonti A., Lenzken S.C., Filosa G., Alvarez R., Calogero R., Bianchi M.E., Barabino S.M.L. (2017). Oxidative Stress Controls the Choice of Alternative Last Exons via a Brahma-BRCA1-CstF Pathway. Nucleic Acids Res..

[82] Jagani Z., Mora-Blanco E.L., Sansam C.G., McKenna E.S., Wilson B., Chen D., Klekota J., Tamayo P., Nguyen P.T.L., Tolstorukov M. (2010). Loss of the Tumor Suppressor Snf5 Leads to Aberrant Activation of the Hedgehog-Gli Pathway. Nat. Med..

[83] Mora-Blanco E.L., Mishina Y., Tillman E.J., Cho Y.-J., Thom C.S., Pomeroy S.L., Shao W., Roberts C.W.M. (2014). Activation of β-Catenin/TCF Targets Following Loss of the Tumor Suppressor SNF5. Oncogene.

[84] Choi S.K., Kim M.J., You J.S. (2020). SMARCB1 Acts as a Quiescent Gatekeeper for Cell Cycle and Immune Response in Human Cells. Int. J. Mol. Sci..

[85] Alimova I., Birks D.K., Harris P.S., Knipstein J.A., Venkataraman S., Marquez V.E., Foreman N.K., Vibhakar R. (2013). Inhibition of EZH2 Suppresses Self-Renewal and Induces Radiation Sensitivity in Atypical Rhabdoid Teratoid Tumor Cells. Neuro-Oncology.

[86] Wilson B.G., Wang X., Shen X., McKenna E.S., Lemieux M.E., Cho Y.-J., Koellhoffer E.C., Pomeroy S.L., Orkin S.H., Roberts C.W.M. (2010). Epigenetic Antagonism between Polycomb and SWI/SNF Complexes during Oncogenic Transformation. Cancer Cell.

[87] Joldoshova A., Elzamly S., Brown R., Buryanek J. (2022). Prometastatic CXCR4 and Histone Methyltransferase EZH2 Are Upregulated in SMARCB1/INI1-Deficient and TP53-Mutated Poorly Differentiated Chordoma. J. Mol. Pathol..

[88] Kadoch C., Crabtree G.R. (2015). Mammalian SWI/SNF Chromatin Remodeling Complexes and Cancer: Mechanistic Insights Gained from Human Genomics. Sci. Adv..

[89] Völkel P., Dupret B., Le Bourhis X., Angrand P.-O. (2015). Diverse Involvement of EZH2 in Cancer Epigenetics. Am. J. Transl. Res..

[90] Knutson S.K., Warholic N.M., Wigle T.J., Klaus C.R., Allain C.J., Raimondi A., Porter Scott M., Chesworth R., Moyer M.P., Copeland R.A. (2013). Durable Tumor Regression in Genetically Altered Malignant Rhabdoid Tumors by Inhibition of Methyltransferase EZH2. Proc. Natl. Acad. Sci. USA.

[91] Stacchiotti S., Zuco V., Tortoreto M., Cominetti D., Frezza A.M., Percio S., Indio V., Barisella M., Monti V., Brich S. (2019). Comparative Assessment of Antitumor Effects and Autophagy Induction as a Resistance Mechanism by Cytotoxics and EZH2 Inhibition in INI1-Negative Epithelioid Sarcoma Patient-Derived Xenograft. Cancers.

[92] Italiano A., Soria J.-C., Toulmonde M., Michot J.-M., Lucchesi C., Varga A., Coindre J.-M., Blakemore S.J., Clawson A., Suttle B. (2018). Tazemetostat, an EZH2 Inhibitor, in Relapsed or Refractory B-Cell Non-Hodgkin Lymphoma and Advanced Solid Tumours: A First-in-Human, Open-Label, Phase 1 Study. Lancet Oncol..

[93] Bai J., Ma M., Cai M., Xu F., Chen J., Wang G., Shuai X., Tao K. (2014). Inhibition Enhancer of Zeste Homologue 2 Promotes Senescence and Apoptosis Induced by Doxorubicin in P53 Mutant Gastric Cancer Cells. Cell Prolif..

[94] Forrest S.J., Al-Ibraheemi A., Doan D., Ward A., Clinton C.M., Putra J., Pinches R.S., Kadoch C., Chi S.N., DuBois S.G. (2020). Genomic and Immunologic Characterization of INI1-Deficient Pediatric Cancers. Clin. Cancer Res..

[95] Ngo C., Postel-Vinay S. (2022). Immunotherapy for SMARCB1-Deficient Sarcomas: Current Evidence and Future Developments. Biomedicines.

[96] Wang D., Quiros J., Mahuron K., Pai C.-C., Ranzani V., Young A., Silveria S., Harwin T., Abnousian A., Pagani M. (2018). Targeting EZH2 Reprograms Intratumoral Regulatory T Cells to Enhance Cancer Immunity. Cell Rep..

[97] Li L., Fan X.-S., Xia Q.-Y., Rao Q., Liu B., Yu B., Shi Q.-L., Lu Z.-F., Zhou X.-J. (2014). Concurrent Loss of INI1, PBRM1, and BRM Expression in Epithelioid Sarcoma: Implications for the Cocontributions of Multiple SWI/SNF Complex Members to Pathogenesis. Hum. Pathol..

[98] Kohashi K., Yamamoto H., Yamada Y., Kinoshita I., Taguchi T., Iwamoto Y., Oda Y. (2018). SWI/SNF Chromatin-Remodeling Complex Status in SMARCB1/INI1-Preserved Epithelioid Sarcoma. Am. J. Surg. Pathol..

[99] Fang R., Xia Q., Wang X., Pan R., Ni H., Wang Z., Rao Q. (2022). Frameshift Mutation and Inactivation of ARID1A in an Epithelioid Sarcoma. Pathology.

[100] Srinivasan A., Liu M., Parham D., Li M., Wang X., Lu X., Li S., Zhang L., Yu Z. (2020). Infantile Epithelioid Sarcoma with Genomic Segmental Amplification of *BIRC3/YAP1* as Double Minutes Plus Trisomy 2: A Case Report. Fetal Pediatr. Pathol..

[101] Patton A., Oghumu S., Iwenofu O.H. (2022). An *SS18::NEDD4* Cutaneous Spindled and Epithelioid Sarcoma: An Hitherto Unclassified Cutaneous Sarcoma, Resembling Epithelioid Sarcoma with Aggressive Clinical Behavior. Genes Chromosomes Cancer.

[102] Cascio M.J., O’Donnell R.J., Horvai A.E. (2010). Epithelioid Sarcoma Expresses Epidermal Growth Factor Receptor but Gene Amplification and Kinase Domain Mutations Are Rare. Mod. Pathol..

[103] Xie X., Ghadimi M.P.H., Young E.D., Belousov R., Zhu Q., Liu J., Lopez G., Colombo C., Peng T., Reynoso D. (2011). Combining EGFR and MTOR Blockade for the Treatment of Epithelioid Sarcoma. Clin. Cancer Res..

[104] Imura Y., Yasui H., Outani H., Wakamatsu T., Hamada K., Nakai T., Yamada S., Myoui A., Araki N., Ueda T. (2014). Combined Targeting of MTOR and C-MET Signaling Pathways for Effective Management of Epithelioid Sarcoma. Mol. Cancer.

